# A Recent Investigation on Detection and Classification of Epileptic Seizure Techniques Using EEG Signal

**DOI:** 10.3390/brainsci11050668

**Published:** 2021-05-20

**Authors:** Sani Saminu, Guizhi Xu, Zhang Shuai, Isselmou Abd El Kader, Adamu Halilu Jabire, Yusuf Kola Ahmed, Ibrahim Abdullahi Karaye, Isah Salim Ahmad

**Affiliations:** 1 State Key Laboratory of Reliability and Intelligence of Electrical Equipment, Hebei University of Technology, Tianjin 300130, China; zs@hebut.edu.cn (Z.S.); isselmou_kader@yahoo.com (I.A.E.K.); karayeb13264@yahoo.com (I.A.K.); isahsalimahmad@gmail.com (I.S.A.); 2 Biomedical Engineering Department, University of Ilorin, P.M.B 1515, Ilorin 240003, Nigeria; ahmed.yk@unilorin.edu.ng; 3 Department of Electrical and Electronics Engineering, Taraba State University, Jalingo 660242, Nigeria; adamu.jabire@tsuniversity.edu.ng

**Keywords:** epileptic seizure, EEG, wavelet, statistical parameters, SVM, random forest, deep learning, disorders of consciousness

## Abstract

The benefits of early detection and classification of epileptic seizures in analysis, monitoring and diagnosis for the realization and actualization of computer-aided devices and recent internet of medical things (IoMT) devices can never be overemphasized. The success of these applications largely depends on the accuracy of the detection and classification techniques employed. Several methods have been investigated, proposed and developed over the years. This paper investigates various seizure detection algorithms and classifications in the last decade, including conventional techniques and recent deep learning algorithms. It also discusses epileptiform detection as one of the steps towards advanced diagnoses of disorders of consciousness (DOCs) and their understanding. A performance comparison was carried out on the different algorithms investigated, and their advantages and disadvantages were explored. From our survey, much attention has recently been paid to exploring the efficacy of deep learning algorithms in seizure detection and classification, which are employed in other areas such as image processing and classification. Hybrid deep learning has also been explored, with CNN-RNN being the most popular.

## 1. Introduction

According to the International League Against Epilepsy, epilepsy is a momentary event of signs and symptoms due to abnormal synchronization and rapid neuronal activities in the brain [1,2]. It is one of the brain neurological chronic disorders that affect around 50 million people worldwide due to the brain cells’ excessive electrical activities, and it is characterized by epileptic seizures [3]. These epileptic seizures can result in neurological, physiological, social and cognitive consequences as a result of loss of consciousness and can even lead to death if proper monitoring and diagnosis have not been in place [4,5].

The loss of consciousness as a result of epileptic seizures has some common features with disorders of consciousness (DOCs), as established in the literature such as the work of [6,7,8]. In this condition, the eyes of the patient may be open, but even with external stimuli, their response might be meaningless. Moreover, a simple response/behavior may be observed even though the presence of sleep–wake cycles cannot be guaranteed due to a lack of sufficient time to determine its presence. Therefore, some of the types of disorders of consciousness exhibited during the occurrence of seizures are acute consciousness disorders (ACDs) that include coma, confusion, drowsiness and stupor, as well as delirium and chronic disorders of consciousness (CDOCs) that consist of minimally conscious and vegetative states (VS) [9]. One major difference between impaired consciousness during the seizure and these types of DOCs is in their duration, in which seizures only last for a short time, with the exception of status epilepticus, while other DOCs last for days, months or years [6,10]. The convergence of some types of DOCs and epileptic seizures to a common structure such as in cortical and subcortical regions helps researchers to develop models that improve epileptic seizure patients’ lives and treatment methodologies by analyzing the behavioral and clinical features of these types of DOCs [11]. The detection, prediction and classification of epileptic seizures may shed more light on determining the pathophysiology and physiology of other types of DOCs. Two types of seizures have been considered from the monitoring aspect: electrographic and behavioral. An electrographic or electroencephalographic epileptic seizure is an irregular paroxysmal pattern of an electroencephalogram (EEG). Simultaneously, a behavioral epileptic seizure is the clinical signs of epilepsy that the patient or an observer can observe or that can be recorded on video [12].

The observation and diagnosis of epileptic seizures manually by a neurologist is tedious, time-consuming and easily prone to errors. The development of an automatic computer-aided system is therefore of paramount importance to help neurologists and patients identify and detect epileptic seizures by minimizing the long-term EEG recording to be analyzed by neurologists [13,14]. To develop an automatic CAD system, there are several steps for epileptic seizure detection from EEG analysis such as signal acquisition, data preprocessing, feature extraction, channel selection, classification and performance analysis/decision making. Due to the complex morphology of the EEG signal and visual similarity between epileptic and normal signals, suitable and meaningful features need to be extracted for classifiers to properly and correctly recognize and characterize different epileptic seizures [15,16,17].

The EEG signals can be used to acquire significant information to describe neurological conditions and need to be recorded to localize epileptic seizures. One of the most important scales in clinical EEGs for evaluating defects and cognition is frequency. A recorded EEG has a frequency somewhere within the 0.01 to 100 Hz range. The frequency content can be divided into five major bands known as delta, theta, alpha, beta and gamma [18,19,20,21,22]. Details on the frequencies associated with these bands are provided in Table 1. The abnormal activities exhibited by epileptic patients are in ictal and interictal conditions. Ictal refers to the epileptic seizure activity, while interictal is the activity that occurs between two epileptic seizures and can be regarded as seizure-free activity. Sharp, spikey, complex and uninterrupted or continuous structural wave forms are usually seen in the ictal signals, while interictal signals are seen as sharp, spikey and temporary waveforms. Research studies [16,22] have shown that some characteristic changes in the EEG signals following a seizure can be detected so that the dynamic mechanisms of the seizures are characterized, identified and localized. An intracranial recording is also conducted in some patients to determine the brain region responsible for initiating the seizure and implantable devices for epilepsy treatment [22,23,24,25].

Researchers have explored different types of methods and domains for automatic seizure detection such as the time domain, frequency domain, time–frequency domain, non-linear methods and Empirical mode decomposition (EMD). However, studies have shown significant improvements in performance when two or more conventional methods are combined [12,26], which describe methods for seizure detection and provide mathematical descriptions of these methods. The authors of [27] provided a review on the applications of entropies with their advantages and disadvantages in epilepsy analysis. A brief description of the epileptic seizure detection and analysis process including preprocessing, feature extraction, feature ranking/selection and classification was conducted in [28]. Automated epileptic seizure detection techniques based on multi-domain approaches were reviewed and highlighted in [29]. In [30], the authors provided a background of pattern recognition in epileptic seizure detection with a review and analysis of some works conducted in epileptic detection, emphasizing analysis of the DWT influence in epileptic detection systems. Focal and non-focal characterization and localization in seizure detection systems were reviewed by [31,32]. Various parameters such as fractal dimension, entropy and Hjorth parameters were used in focal and non-focal EEG signal characterization, and their performances were compared using the Bern-Barcelona EEG database.

Most of the review articles found in the literature on epilepsy detection systems are focused on conventional or traditional techniques. However, recently, much attention is being paid to machine learning and, now, deep learning networks to explore their potential in the detection and characterization of epileptic seizures. Therefore, this study highlights various techniques for feature extraction and selection commonly used in epileptic seizure recognition systems in conventional methods and deep learning from 2010 to 2020. It also includes the fundamental components of an EEG seizure detection system and performance metrics.

This paper reviews the classification techniques commonly used in epileptic seizure detection. Our review includes works that used EEG and intracranial EEG (iEEG) or both in their seizure detection models. Reliable and significant feature extraction methods were investigated. A comparison of the performance of various algorithms for the recognition of seizures and classification systems were explored and analyzed. This work will bring researchers up to date on the significant feature extraction techniques, statistical and machine learning classifiers and recent deep learning algorithms. Another contribution of this review is to help researchers to identify publicly available databases of recorded epileptic seizure signals. Finally, based on this current review, suggestions on future research directions are provided.

## 2. Epileptic Seizure Detection System

**Figure 1:**
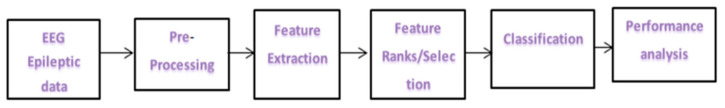
Block diagram of an epileptic seizure detection system.

This section provides a general overview of an epileptic seizure detection system. A typical system consists of the following stages, as shown in Figure 1: 1. data acquisition, 2. preprocessing, 3. feature extraction, 4. classification and 5. performance analysis and evaluation.

### 2.1. Data Acquisition and EEG Database

The study of epileptic seizure detection and analysis has been carried out with both scalp EEG recordings (EEG) and intracranial EEG recordings (iEEG). Scalp EEG recordings use electrodes placed on the surface of the head at equal distance with the 10–20 system as the most commonly used configuration [20,33]. The iEEG signals use intracranial electrodes placed inside the skull when the clinical, structural and functional data are obtained before implantation to locate the epileptogenicity region in the brain [22].

Local databases that were used in previous studies were developed based on the information and data obtained and analyzed from epilepsy patients before epileptic surgeries. The small sample sizes, short time durations prior to seizures and small seizure actions hindered their applicability, limiting the specificity evaluation in the interictal signals. Therefore, recording of long-term signals from various seizures to properly and efficiently evaluate the sensitivity and specificity of algorithms is necessary [32].

**Figure 2:**
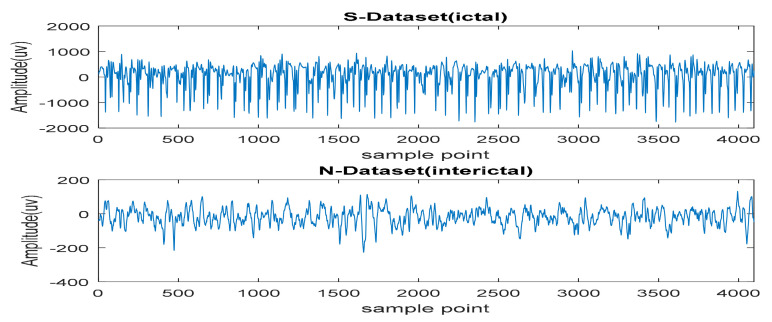
Example of epileptic seizure signals for ictal and interictal conditions.

Recently, various research works on epilepsy have employed some online databases that are publicly available, while some require permission from the owners such as the Andrzejak database [34] from the Department of Epileptology, University of Bonn, Germany, the Freiburg database from the Epilepsy Centre of the University Hospital of Freiburg, Germany [35], the Boston Children’s Hospital-MIT EEG datasets [36] and the Bern-Barcelona database from the University of Bern, Barcelona, Spain [37]. The largest epileptic seizure database available is the European Database on Epilepsy, with 2500 recorded seizures in 45,000 h of recording duration. Among the more than 250 subjects, 50 underwent iEEG at a sampling frequency of 250–2500 Hz over 122 channels [38]. Another recently used database is the data obtained from the Neuro Vista ambulatory monitoring system, which supplied continuous iEEG signals for many months [39,40]. Figure 2 shows University of Bonn data for class S for ictal conditions and class N for interictal conditions.

### 2.2. Preprocessing

Biomedical signals are usually contaminated with various types of noise and artifacts during data acquisition and processing, which greatly influences the quality of feature extraction techniques. The artifacts’ sources are generally categorized into technical, physiological and environmental sources [41,42,43]. Therefore, one of the aims of biomedical signal processing is to search for how to minimize or eliminate artifacts and still retain the most useful and relevant information in the raw EEG signal.

Artifacts that are caused by technical issues or instruments used during EEG acquisition are related to the equipment’s settings and the EEG type, that is, either an EEG recorded from the scalp or an intracranial EEG recording [17]. Some of these settings are gain, high-pass and low-pass filters’ cut-off frequencies, sampling rate and electrode types. Artifacts due to physiological sources are electromyograms (EMGs), which are muscle activity, electrooculograms (EOGs) due to eye movement and electrocardiograms (ECG), which are due to the heart rate activity. In contrast, environmental interference depends on the environmental conditions and setting of EEG acquisition and recording [44,45,46]. Artifacts can be divided into two groups: physiological and nonphysiological artifacts, as summarized in Table 2.

The presence of these strong unwanted components severely reduces the quality of the signal and diminishes the accuracy of further processing such as feature extraction and classification. Therefore, the need for denoising and removing these artifacts and noises can never be overemphasized. Different techniques and algorithms have been developed to eliminate artifacts and noise to make the process more reliable for further processing and analysis [45]. These methods include early prevention steps taken during the EEG recording, which include preventing muscular and ocular artifacts by limiting eye blinking, eye movements and movements by other parts of the body. Another method is the threshold criterion which excludes corrupted trials of EEG signal recordings [47]. The method of location and elimination of contaminated activity is also a feasible approach using the electrooculogram (EOG) subtraction technique [48,49]. Independent component analysis (ICA) is one of the most popular techniques in EEG artifact rejection and denoising with excellent results. Researchers have extensively studied time–frequency techniques as a viable approach that includes wavelet transform denoising techniques. The autoregressive method, proposed in [50], can be used to subtract the artifact signal from the original EEG signal. Adaptive filtering is also another technique to optimize performance by adjusting its transfer function by itself. Other techniques include the support vector machine (SVM) approach, which categorizes the EEG epilepsy signal into different classes and then eliminates artifacts such as head movement [51].

#### 2.2.1. Filtering Technique

One of the popular techniques for artifact elimination/reduction is the filtering technique. In the filtering technique, filters are applied to the raw EEG signals to remove or reduce artifacts and noise for better EEG interpretation, diagnosis and analysis. The filtering technique has been used in EEG signals for removing power line noise (50 Hz or 60 Hz), unwanted high-frequency components such as artifacts generated from muscular activities and low-frequency components such as low-frequency drifts.

Several filtering approaches have been developed by researchers over the years, from simple classical approaches [52,53] to adaptive approaches [54,55]. The Kalman filter, Weiner filter and Bayes filters are some of the common filtering approaches [29]. However, adaptive filtering has the best performance. Adaptive filtering, unlike the simple filtering technique that uses a fixed frequency range, adaptively adjusts its weights after estimating the artifact signals using a reference signal, and a clean EEG signal is obtained after subtracting the estimated artifactual components. It is easy to use, has no calibration requirements and can be implemented online. These are some of its advantages. However, the use of reference signals in this technique requires additional sensors, which increase the cost and complexity.

**Figure 3:**
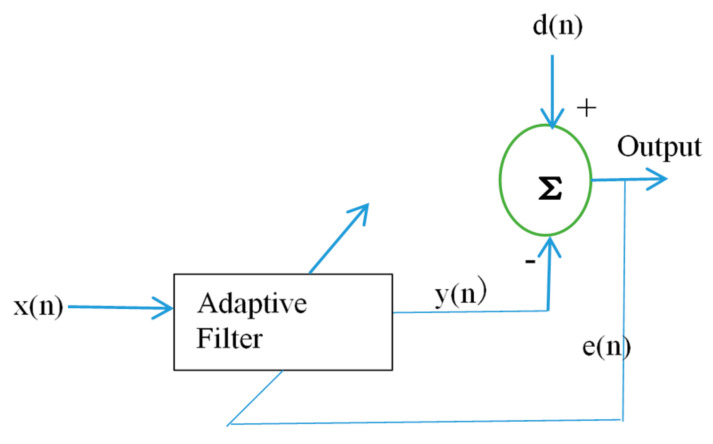
Adaptive filtering method.

The structure of adaptive filtering is shown in Figure 3, where $d\left(n\right)$ is the desired signal, $x\left(n\right)$ is the reference signal, *y*(*n*) is the adaptive filter estimated output and $e\left(n\right)$ is the residual error which is given in Equation (1):

(1) $$e\left(n\right)=d\left(n\right)-y\left(n\right)$$

Adaptive filtering uses optimization algorithms to help in adjusting its weights to obtain the optimum filter coefficients. Recursive least squares (RLS) is one of the best and common optimization algorithms employed in adaptive filtering [56]. The least mean squares (LMS) algorithm is another optimization algorithm used in the adaptive filtering technique [57,58,59].

#### 2.2.2. Blind Source Separation Techniques

Blind source separation techniques (BSS) are some of the most commonly used techniques for artifact and noise removal from EEG data by excluding neuronal activity signal sources from artifact source signals [60,61,62,63]. One of the major merits of BSS is that the previous mixing information from different sources is not needed, or in some situations, a very small amount of information is needed. Let *X* be multi-channel EEG signals with linear mixing of sources *S*; then, mathematically,

(2) X=AS

where *A* is the mixing matrix. BSS can be used to generate an un-mixing matrix *W* to separate the sources:

(3) $$\hat{S}=WX$$

where $\hat{S}$ is the estimation of the sources.

Once all of the neuronal and artifactual sources are known, the latter can be removed to obtain an artifact-free EEG. There are many BSS algorithms developed to remove artifacts from EEG signals, including independent component analysis (ICA), principal component analysis (PCA), canonical correlation analysis (CCA) and morphological component analysis (MCA).

In the preprocessing stage, a signal is also normalized to compare the signal with that of different patients and that recorded by another acquisition system.

## 3. Feature Extraction Techniques

To develop a robust automated scheme for epileptic seizure detection, categorizing EEG signals (epileptic seizures) into a pre-seizure, seizure and post-seizure occurrence must be identified and evaluated. Many features have been explored in the literature to describe seizure behavior properly. These features describe the EEG static behavior in time and space as well as dynamic properties. Feature extraction techniques commonly found in the literature include time domain, frequency domain and time–frequency analyses, wavelet analysis, energy distribution, entropy analysis and feature tensors [64]. However, recently, most CAD systems use two or more methods combined as a hybrid technique.

### 3.1. Time Domain Analysis

Epileptic EEG signals in their raw form are a function of time. Therefore, features that are calculated and extracted on these signals are called time domain features, although time domain features are not mostly used alone in EEG epileptic signal analysis. Some features such as amplitude, synchronization and regularity, which change during epileptic seizure events, characterize the EEG signal. Some of the works that used these features include [65,66,67], in which the relative duration, relative average amplitude and the coefficient of amplitude were used in epileptic seizure detection techniques. Another method is to use empirical mode decomposition (EMD); this method is applied to nonstationary signals in nature [68]. Several works have reported accuracies obtained after applying higher-order spectra in their approach, as in [69,70,71]. This paper selected some feature extraction techniques in the time domain that are predominantly new in the literature and explained as follows.

#### Statistical Parameters

Researchers have used statistical parameters such as skewness, kurtosis and line length to characterize between non-seizure and seizure conditions because the statistical distribution of EEG signals for various conditions is different. Therefore, these parameters are calculated as features to differentiate between normal and a seizure event.

For example, let *X* be the sequence used for feature extraction such as an epoch of an EEG signal, donated as in Equation (4) [65]:

(4) X=[x[0],x[1]……x[N−1]]

where *N* is the length of the sequence.

The most common statistical parameters used in extracting features are as follows:

(5) $$mean=\frac{1}{n}\overset{n}{\underset{1}{\sum }}x_{i}$$

(6) $$median={\left(\frac{N+1}{2}\right)}^{th}$$

(7) $$S.D.=\sqrt{\sum_{1}^{n}\left(X_{n}-mean\right)}\frac{2}{n-1}$$

(8) $$skewness=\sum_{n=1}^{N}\left(x_{n}-mean\right)\frac{3}{(N-1)S.{D.}^{3}}$$

(9) $$kurtosis=\sum_{n=1}^{N}\left(x_{n}-mean\right)\frac{4}{(N-1)S.{D.}^{4}}$$

(10) $$\max=\max[x_{n}]$$

(11) $$\min=\min[x_{n}]$$

The curve length or line length is expressed as

(12) $$L(x)=\sum_{i=1}^{N}\left|x[i]-x[x-1]\right|$$

Other statistical variants include average power, energy, root mean squared value (RMS), cross-correlation, independent component analysis, linear discriminant analysis and principal component analysis, among others.

### 3.2. Frequency Domain

To capture the frequency components of epileptic EEG signals during various signal seizure conditions, signal transformation is conducted to describe the details of the frequency representation of the signal to obtain some useful information about the signal. The popular Fourier transform calculates all the frequency components in the signal so that different brain activities can be isolated and described based on their frequency. To extract features based on the signal power division at each frequency, the power spectral density (PSD) method is used to calculate and analyze the features. Some of the spectral features calculated using the PSD technique include peak frequencies or dominant frequency, average band frequency, spectral edge frequency, intensity weighted bandwidth and the bandwidth of the dominant frequency [72].

One of the methods for obtaining the PSD values is using a Welch frequency estimation technique. The EEG data are segmented into overlapping segments, and each segment is windowed, averaged and estimated from its periodogram.

If *x*(*n*), wherein *n* = 1,2, …, *N*, is the data sample derived from the available signal data, the estimated periodogram is given as [32]

(13) $${\hat{P}}_{PER}\left(f\right)=\frac{1}{N}{\left|\sum_{n=1}^{N}x\left(n\right)e^{-i\omega fn}\right|}^{2}$$

where ${\hat{P}}_{PER}\left(f\right)$ is the periodogram power estimation. If the data segments are expressed as *x_l_* (*n*), *l* = 1,2, …, *S*, the Welch spectrum is given as

(14) $${\hat{P}}_{w}\left(f\right)=\frac{1}{S}\sum_{l=1}^{S}{\hat{P}}_{l}\left(f\right)$$

(15) $${\hat{P}}_{l}\left(f\right)=\frac{1}{M}\frac{1}{P}{\left|\sum_{n=1}^{M}v\left(n\right)x_{l}\left(n\right)e^{-i\omega fn}\right|}^{2}$$

where *M* is the length of each EEG segment, while ${\hat{P}}_{l}\left(f\right)$ is the periodogram estimation of the first segment, *v*(*n*) refers to a data window, ${\hat{P}}_{w}\left(f\right)$ denotes Welch PSD values, *S* refers to segment number and *P* is the average of *v*(*n*), which is expressed as

(16) $$P=\frac{1}{M}\sum_{n=1}^{M}{\left|v\left(n\right)\right|}^{2}$$

This approach is known as the non-parametric method, and its limitation is spectral leakage due to its windows. The parametric method is proposed to overcome non-parametric limitations. The signal is taken as a random stationary process, with the noise as input when the signal is modeled as filter output. Filter parameters are later determined after that. One of the parametric methods is the autoregressive model. This technique uses a linear combination of the signal’s earlier activities with uncorrelated noise [26,73,74], given as

(17) $$e_{i}=\sum_{j=0}^{P}A_{j}x_{i-j}$$

where *Xi* is the input signal, *Aj* is the model coefficient matrix, *p* is the model order and *e_i_* refers to a multivariate zero-mean uncorrelated vector.

(18) $$\sum_{j=0}^{p}A_{j}R(j-k)=-R(-k),k=1,\ldots ,m$$

To determine the *A_j_* matrix, the linear equation *m x p*: was solved, where *m* is the number of channels, *p* is the AR model’s calculated order and *R*(*k*) refers to the covariance matrix biased values.

In [75], the authors applied a step-wise least square estimation algorithm (SLSA) on seizure and normal EEG signals to estimate the autoregressive model (AR) orders. In contrast, the Burg method was applied for the estimation of PSD values. EEG epileptic seizures were classified with the SVM method based on an optimal AR model order and firefly optimization (FA) [76].

### 3.3. Time–Frequency Domain

The shortcoming of time domain analysis is that, while the exact location of events can be located, the events’ frequency components cannot be determined. While the frequency domain provides information on frequencies involved in the signal, it cannot provide information about when they occur. The time–frequency domain was developed to overcome the limitations of the time domain and frequency domain. Several techniques for signal transformation and decomposition to provide information in both time and frequency have been developed in the literature [12]. Short-time Fourier transform (STFT), Weiner–Ville distribution (WVD), spectrography and wavelet transform analysis are commonly used techniques to calculate and extract epileptic EEG features.

### 3.4. Wavelet Analysis

Wavelet transform (WT) is a popular biomedical signal processing approach due to its oscillatory nature, finite length and suitability in dealing with nonstationary and transient biomedical signals [77,78]. In EEG epileptic seizure signal analysis, WT is used to decompose the signals into various components by using scaling and shifting functions over the whole signals to obtain a signal component in time and frequency domains simultaneously [79]. Mother wavelet has to be chosen as a function that can be used to interpret the original signal into sub-bands. Generally, wavelet functions can be defined in Equation (19) as

(19) $$\Psi_{s,\tau }=\frac{1}{\sqrt{s}}\Psi \left(\frac{t-\tau }{s}\right)$$

where *s* is the scale parameter and τ is the shift parameter.

From Equation (19), wavelet transform is given as

(20) $$\gamma \left(s,\tau \right)=\int f\left(t\right)\Psi_{s,\tau }^{*}\left(t\right)dt$$

Meanwhile, Equation (21) defines inverse wavelet transform as

(21) $$f\left(t\right)=\iint \gamma \left(s,\tau \right)\Psi_{s,\tau }\left(t\right)d\tau ds$$

**Figure 4:**
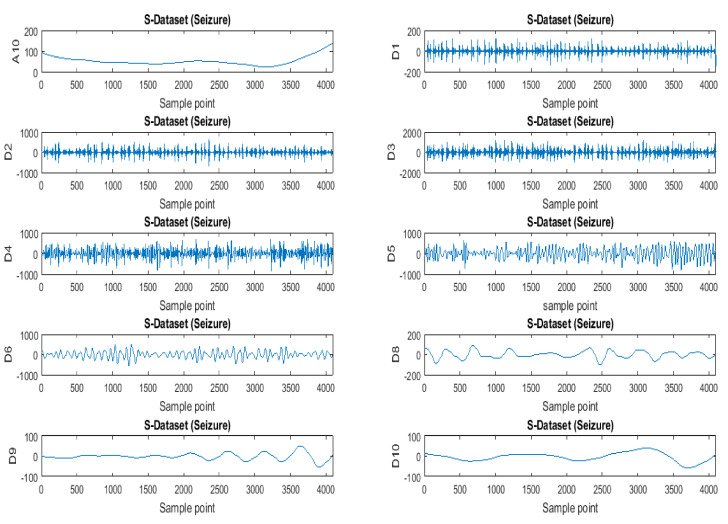
Discrete wavelet decomposition.

In discrete wavelet transform (DWT), a low-pass filter *g*[*n*] and a high-pass filter *h*[*n*], which correspond to scaling and shifting functions, respectively, were designed [80] as quadrature mirror filters successively. These filters produced approximation coefficients and detail coefficients, as shown in Figure 4. The decomposition level should be chosen so that the filtering and decimation processes continue up to that level [81,82].

Let an EEG signal be *x*(*n*), decomposed into multiple frequency bands of different scales (*j*), and assume the length of the signal *N* satisfies Equation (22):

(22) $$N=2^{j}$$

**Figure 5:**
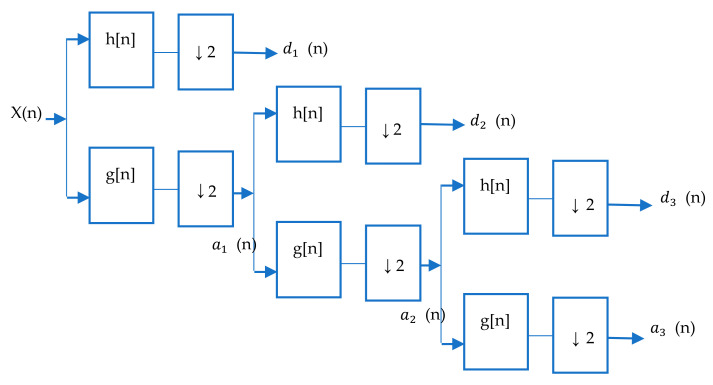
Example of epileptic seizure signal decomposed into various levels.

The decimation process is performed at each level by downsampling the frequency by half to obtain a good frequency resolution. The efficacy of wavelet transforms in EEG epilepsy detection analysis has been explored by many research works. Figure 5 shows an example of an epileptic seizure signal from the Bonn University dataset decomposed up to level 10. The detail coefficients, which contain most of the noisy components, are set to zero as most of the signal information lies in approximate coefficients (low frequency). This process is also known as thresholding. However, the authors of [72,73,74,75] addressed the limitation associated with thresholding (i.e., deciding thresholding values for detail coefficients to be chosen).

Most of the works reported in the literature that used wavelet analysis combined this approach with another technique.

Other techniques employed and used in time domain analysis are statistical param-eters, curve length or line length, energy, power and RMS. According to Logesparan et al. [83], line length is one of the best features for characterizing the epileptic EEG region.

### 3.5. Non-Linear Analysis

The non-linearity of EEG epileptic signals can be well detected by the frequency domain. The non-linearity and non-stationarity of EEG signals render it to be considered chaotic. Therefore, the changes in EEG signals are difficult to be detected by visual inspection [84]. Common techniques to detect minute data changes due to EEG signals’ non-linear and dynamic behavior are entropies and Lyapunov techniques.

#### Entropy Analysis

Entropy may generally be defined as the measure of uncertainty and fluctuation of a system. The values of entropy represent the degree of uncertainty and how chaotic a system is. Larger values of entropy indicate a more chaotic and uncertain system. Various entropy estimators have been applied to detect and analyze EEG epileptic signals to distinguish and inspect seizure occurrence and normal signals. The most common entropy estimators are Shannon entropy, approximate entropy, sample entropy, Renyi’s entropy, fuzzy entropy and permutation entropy [85,86,87,88].

## 4. Classification Techniques

The quality of classification algorithms is largely dependent on the feature extracted and fed to the classifier. The features are extracted with the assumption that they can be characterized between normal and different seizure categories. Classifiers are decision-making systems in which the class data boundaries are defined and labeled based on their features. The classification method can be simple such as thresholding techniques, or complex such as machine learning algorithms.

In the classification stage. There are generally two steps to be carried out, that is, training and testing phases. The extracted features are divided into those phases, and after training the classifier with training data, the new data can be classified with the trained network. Classifiers in epileptic seizure detection systems can be developed using statistical analysis such as clustering, machine learning or, recently, deep neural networks [89].

### 4.1. Machine Learning Techniques

Machine learning algorithms are the most widely used classifiers in automated epilepsy detection systems. The conventional handcrafted feature extraction methods are used to extract features and statistically analyze, rank and select data that are used as input to machine learning algorithm classifiers. Several classification techniques have been proposed in the literature, such as k-nearest neighbor (k-NN), logistic regression, random forest, artificial neural networks (ANNs), fuzzy logic and SVMs with various kernel functions. A list of studies using machine learning algorithms with different feature extraction techniques is shown in Table 3.

Table 3 compares the performance of various EEG detection algorithms in past works in terms of the feature extraction technique, the classifier employed and the accuracy obtained. Faust et al. [90] achieved an accuracy of 98.33% using a single-feature, PSD and radial basis function SVM model. An accuracy of 100% was recorded with the DWT feature by Upadhyay et al. [109] with an LS-SVM model. A new feature, Teager energy, was used by Sriraam et al. [124] to achieve 96.66% accuracy with supervised backpropagation. Some studies have also employed multiple features with an ML technique such as the work of Saminu et al. [144]. This study used a feedforward neural network (FFNN) coupled with an SVM to detect and classify ictal and interictal signals. It was computationally less complex with a high accuracy of 99.6%. Mahjoub et al. [145] conducted feature extraction of epileptic EEGs with tunable-Q wavelet transform (TQWT) and intrinsic mode functions (IMFs) of multivariate empirical mode decomposition (MEMD) and directly from the EEG raw data. This approach was a mix of linear and non-linear parameters and multiple features as its edge; an accuracy of 98.7% was recorded with SVM. From Table 3, it can be seen that the genetic algorithm, Bayesian net and fuzzy clustering are not popular classifiers in EEG signal processing [101,105,111]. RFC, ANN and KNN are quite promising classifiers with great accuracy [116,146,147]. However, SVM is the most commonly applied classifier [116,117,118,119,120,121,122,123,124,125,126,127,128,129,130,149,150,151,152].

#### Overview of Support Vector Machine

**Figure 6:**
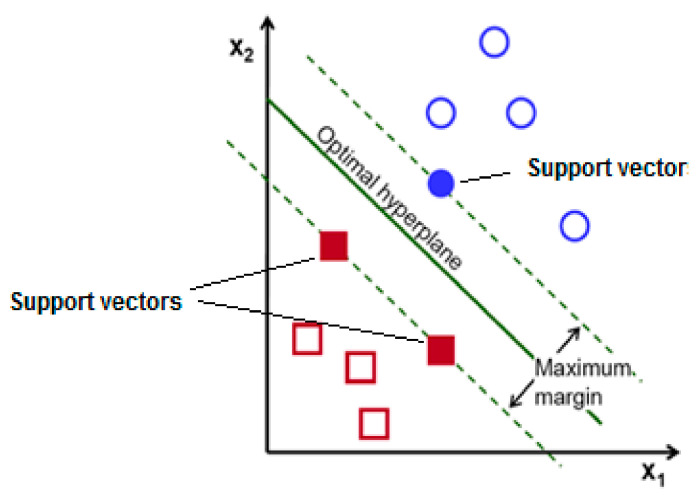
An example of a separable problem in a 2D space.

SVM is a machine learning classifier highly suitable for binary classification with feature vectors of a high dimension. It is very suitable and popularly used in biomedical signal processing and applications due to its capability to deal with many predictors and high accuracy. The distance of the optimal hyperplane obtained by SVM from the feature space of a high dimension and that of each class closest to the data sample is maximized by SVM [153]. It depends on its regularization parameter, which controls the level of overlap between the class and kernel functions, which is used to map training data to a feature space of a higher dimension from an input space [154]. Figure 6 depicts an example of a 2D separable classification problem by denoting the maximum margin and optimal hyperplane. The support vectors are those data points on the margin line [155].

One of the common kernel functions used in SVM is the linear kernel function with the following equation:

(23) $$K\left(X,Y\right)=X^{T}Y$$

Another type of kernel function is a polynomial with a degree d as follows:

(24) $$k\left(X_{i},X_{j}\right)={\left(X_{i},X_{j}\right)}^{d}$$

where $d\left(d\geq 1\right)$ is the number of polynomials.

If the number of polynomials is d=2 or d=3, then the function is called a quadratic kernel function.

### 4.2. Deep Learning Techniques

Deep learning algorithms were employed in automated epilepsy detection systems to cater for the limitations associated with machine learning techniques. DL does not require handcrafted features to be extracted manually; due to its multilayer architecture, it can deal with large datasets, execute imbalanced datasets and provide a result without biasing towards a majority class [156,157]. Some of the DL architectures include long short-term memory (LSTM) networks, convolutional neural networks (CNNs) and gated recurrent units (GRUs). A variety of convolutional models have been proposed and applied by different researchers to investigate their capability in automated epilepsy detection systems [158,159,160].

The most common approach is a convolutional neural network with a variety of architectures such as temporal CNNs (TCNNs), temporal graph convolutional networks (TGCNs) and CNN-recurrent neural networks (RNNs) [161]. A CNN’s basic structure consists of convolutional layers, max pooling layers, fully connected layers and softmax layers [162,163].

Uniquely, the CNN architecture conducts feature extraction automatically by itself in the process of classifying the EEG signal. The convolutional layer conducts the filtering/feature extraction, while the max pooling layer carries forward the significant feature decided/chosen by the convolutional layer. The fully connected layer simply compiles the extracted data for the softmax layer that conducts the binary classification, i.e., converting the data into probabilities between 0 and 1.

Although deep learning algorithms outperform their conventional counterparts, large datasets’ requirements for their operation become their major limitation. A list of works in automated epilepsy detection and analysis that used deep learning methods is summarized in Table 4.

Table 4 compares the performance of various EEG detection and classification algorithms previously employed by researchers using deep learning schemes. Gao et al. [189] recently implemented a deep convoluted neural network (DCNN) for epileptic EEG signal classification called (EESC). They used PSD energy diagrams for feature extraction with accuracy of over 90% on the CHB-MIT EEG dataset. Jang and Cho [180] proposed a dual deep neural network using spectral analysis features for automatic detection of seizures from EEG signals. It has a low computational cost and a sensitivity of 100%. In focal epileptic seizure detection, a CNN (1D and 2D) and/or LSTMs were adopted by Tjepkema-Cloostermans et al. [174] with an AUC of 0.94 and specificity of 99.9%. A feature learning scheme using unsupervised deep convoluted neural networks proposed by Yuvaraj et al. [175] achieved a sensitivity of 86.29% and a latency rate of 2.1 s. In another study, Nogay et al. [159] implemented a pretrained 2D AlexNet CNN coupled with transfer leaning to detect epileptic seizures from EEG data. It also uses spectrogram short-term images and achieved 100% accuracy. Other studies that used spectrograms include [153,158] and [176]. A 3D kernel of Wei et al. [169] combined 2D images of an individual-channel EEG time series to obtain a 3D image. This was used to predict ictal, pre-ictal and interictal periods with an accuracy > 90%, and sensitivity of 88.9%. Olokodana et al. [160] proposed a DNN with distributed kriging-bootstrapping for seizure classification. This approach achieved relatively high accuracy at 91% in less time than basic DNN. Yuan et al. [170] developed stacked sparse denoising autoencoders (SSDA) for feature extraction from an EEG spectrogram synthesized from short-time Fourier transform. A study by [184] implemented a 2D scalogram derived from continuous wavelet transform for feature extraction. A CNN was used to classify the features over five classes of EEG records, and an accuracy of >90% was obtained across the board.

## 5. Discussion

Selecting the most relevant and significant features is an important step in developing reliable and precise models. Therefore, understanding signals’ statistical properties is crucial as each implanted electrode’s statistical measures and channel are different. Analyzing these properties, such as skewness, energy and entropy, will help researchers avoid using irrelevant features that may increase the dataset and increase the computational complexity of the machine learning classifiers. Most of the researchers adopted testing different machine learning classifiers and evaluated their performance compared to classifiers. The best classifier is considered for brain datasets to solve seizure detection problems.

Several classifiers have been tested and evaluated for EEG epileptic seizure detection to discriminate between seizure and non-seizure states. The heterogeneity of features supplied to classifiers, differences in processing techniques and patient data makes it difficult to compare classifiers. ANN and SVM classifiers are the most common techniques, with the latter being easier and faster than the former.

**Figure 7:**
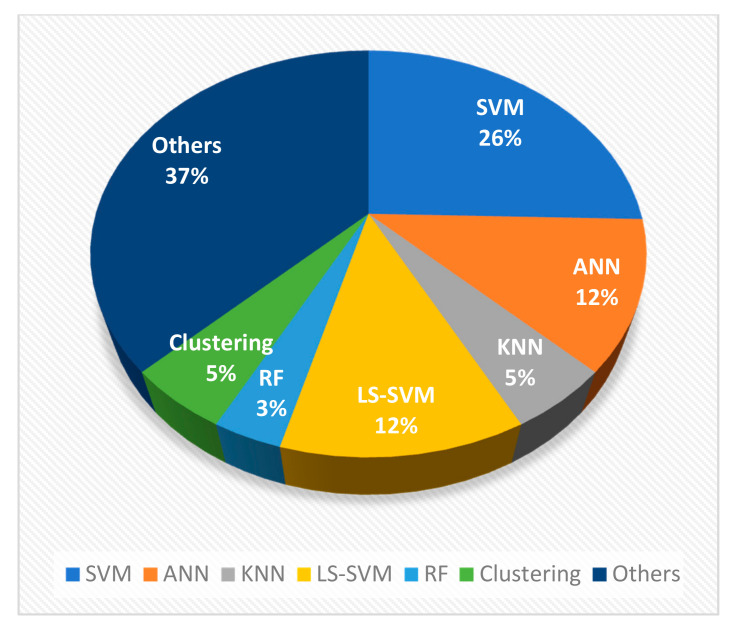
The percentage of conventional techniques involved in epilepsy studies.

**Figure 8:**
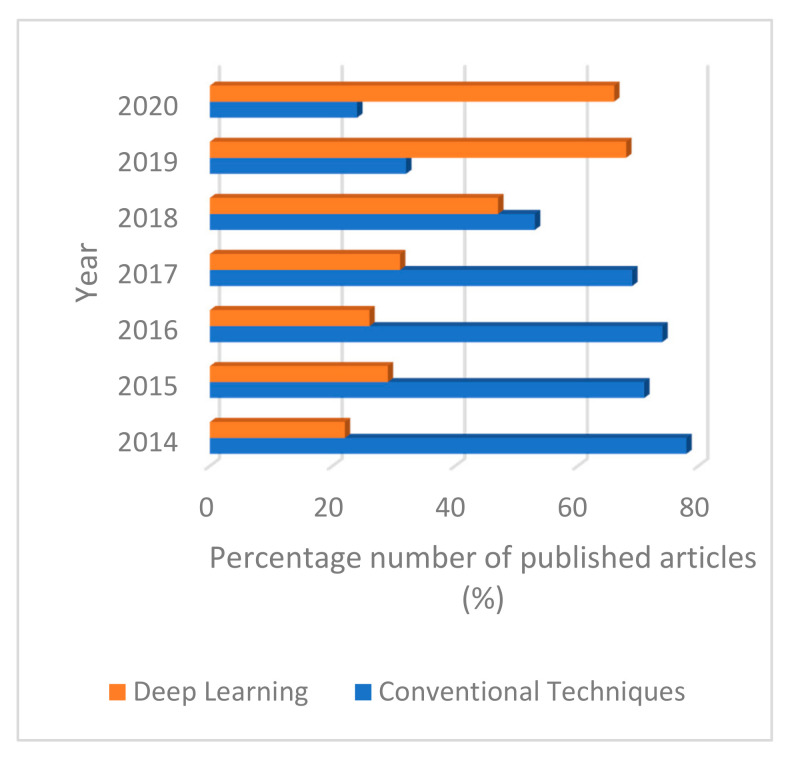
Comparison of conventional techniques and deep learning models used by researchers from 2014 to 2020.

Despite researchers’ contribution and effort to develop and improve seizure prediction and characterization algorithms, the realization of clinical devices by converting these existing techniques has been a major bottleneck. Based on the algorithms’ studies, it is evident that the specific build-up to a seizure state is responsible for the seizure and not a random process. From this review, most researchers employed feature extraction schemes such as wavelet transform, statistical methods and chaos techniques such as entropy analysis. However, in deep learning EEG seizure application, periodograms are the most promising feature extraction technique. From this survey, it appears that multi-feature extraction schemes did not perform better than single-feature classifiers. Hence, only significant features should be included to avoid increasing complexity with little or no improvement in performance. Wavelet transform combined with other techniques such as entropy and statistical parameters has also been employed [64]. Figure 7 shows the percentage of conventional methods used by researchers based on our reviewed articles’ analysis, while Figure 8 depicts the comparison of conventional techniques and deep learning models in percentages employed by researchers from 2014 to 2020.

Standardization of epileptic seizure techniques is also an issue of concern because homogenous comparison performance measures must be grouped to provide a homogeneous and standard comparison. Another issue is related to recording the EEG signals’ duration in either scalp EEG or intracranial EEG.

Researchers have devoted much attention to investigating and developing hybrid models over the years, as indicated in Figure 7. The figure shows the percentage of conventional techniques reviewed in this paper. Hybrid techniques are the most employed approach, with 37% of the cases. SVM is the most used technique in the case of stand-alone techniques, which covered 26% of the reviewed articles. Its simplicity, suitability for binary classification, capability to deal with many predictors and high accuracy are some of the advantages of SVM. ANN covered 12% of the reviewed articles which used the number of neurons and layers instead of kernel functions as in SVM. Other techniques investigated are clustering with 5% of the reported articles and the random forest technique with 3% of the investigated techniques.

Figure 8 shows the number in percentage of published articles from 2014 to 2020 for conventional techniques and deep learning approaches. The trends indicate the surge in researchers’ attention towards deep learning approaches from 2014 upwards, with 22% of reviewed articles in 2014, increasing to 68% in 2019. In comparison with conventional schemes, the chart shows a continuous decrease from 78% of published articles in 2014 to 24% in 2020. This shows how researchers have focused their attention on exploring the efficacy of deep learning approaches.

### 5.1. Challenges

Despite the progress achieved in the detection and classification of epileptic seizures recently, there are still some challenges holding researchers back that include, among others, the following: (1) Many research studies have used various available datasets; however, combining these datasets is quite difficult as each has a different sampling frequency, a different number of electrodes and different parameters, which hinders researchers in combining different datasets to obtain a large dataset for training the model. (2) Real-time signals need to be used for detection and classification to realize real-world applications in a clinical setup. Still, most of the datasets available contain a chosen segment of EEG signals that are not suitable for real-world clinical implementation. (3) The lack of standardization among the developed algorithms is another challenge that makes a homogenous performance comparison difficult. (4) In recent deep learning models, the requirement of higher computational resources that are not available to some researchers hinders the realization of reliable, practical and precise non-invasive models that meet the demand of mobile health and IoMT.

### 5.2. Future Research Direction

This paper provides a comprehensive investigation of epileptic seizure identification and detection techniques. Over the years, tremendous progress has been witnessed, ranging from traditional techniques to the recent deep learning application. However, some challenges have been identified and raised that bring some interesting research questions that still need to be addressed to implement and improve these developed models successfully. The following are some of the suggestions for uplifting future research.

1. With a large volume and high dimension of epileptic seizure datasets, dimensional reduction techniques that reduce the dataset dimension and still retain the significant signal information need to be further investigated.
2. Suitable features that reduce the classifier’s computational complexity and time should be considered.
3. For models that use invasive recordings, the developed methods must identify seizure onset and measure the seizure strength.
4. Researchers should choose a classifier that will not miss or skip all the relevant EEG channels and electrodes.
5. Deep learning structures must be carefully selected based on the problem’s peculiarities and involve relevant datasets for real-time, online and offline detection.
6. Hybrid deep learning techniques should be extensively explored.
7. EEG signal analysis is a neurophysiological approach which holds great potential for enhanced diagnosis and classification of acute disorders of consciousness (ADOCs) such as a vegetative state (VS) and a minimally conscious state (MCS), among others. It can be used to predict the dynamics in the thalamocortical connections as it depicts changes in the activities of the reticular system. Detection and classification of epileptic seizures using EEG signals are a significant step towards advanced diagnosis of unresponsive wakefulness syndrome (UWS) and MCS by characterizing the level of awareness as they share some common features with epileptic seizures. Previous work such as that of Naro et al. [193] used γ-band transcranial alternating current stimulation (tACS) as a non-invasive neurostimulation protocol on DOC patients to differentiate UWS and MCS individuals. Another neuromodulation approach was also applied in [194], while electrophysiologically based approaches were discussed in [195]. Further research on deep learning techniques could be employed in the classification of VS, MCS and UWS.

## 6. Conclusions

This study investigated and reviewed various automated EEG epileptic seizure detection and classification techniques. It also highlighted both traditional feature extraction techniques and statistical and machine learning classifiers. Any developed model must be subjected to a rigorous performance evaluation to test its efficacy in identifying and detecting epileptic seizure signals. Conventional feature extraction techniques commonly employed by researchers are wavelet transform, entropy and non-linear techniques. ANN, SVM and random forest are the most commonly used machine learning classifiers, while CNN is most commonly used for deep learning. Further investigation must be thoroughly conducted on seizure detection techniques to improve the outcome. Recent studies have also focused on hybrid deep learning schemes. This recent research direction needs to be investigated and compared with conventional techniques. Advanced detection and classification using EEG signals must be further investigated to characterize the level of awareness in epilepsy and DOC patients to differentiate between VS, MCS and UWS. With all these, the future is very promising for early diagnosis and treatment of epileptic seizures.

## Figures and Tables

**Table 1:** EEG frequency bands.

Frequency Band Name	Frequency Bandwidth (Hz)
Alpha	<4
Beta	4–8
Gamma	8–12
Delta	12–30
Theta	<30

**Table 2:** Types of artifacts in EEG signals.

Interior Artifacts	Exterior Artifacts
Blinking of the eye (EOG)	Power line
Heartbeat (ECG)	Machine fault
Muscle movements (EMG)	Faulty electrode/poor placement
Skin resistance	ventilation
Subject’s movement	Digital artefacts (loose wiring, etc.)

**Table 3:** Summary of reviewed works that used conventional feature extraction techniques and machine learning classifiers.

Author	Year	Features	Classifier	Performance (%)
[90] O. Faust et al.	2010	PSD	RBF SVM	Acc = 98.33
[91] Subasi et al.	2010	PCA, LDA, LDA	SVM	Acc = 98.75
[92] Guo et al.	2010	DWT	ANN	Acc = 99.60
[93] Oweis	2011	EMD + MEMD	Euclidean Clustering	Acc = 94.00
[94] Orhan et al.	2011	DWT	K-Means Clustering	Acc = 96.67
[95] Yuan et al.	2011	Entropy/Hurst exponent	ANN/PD	Acc = 96.50
[96] Marcus and Dragan	2012	Bilinear TFD	SVM/	Acc = 99.30
[97] Arslan et al.	2013	SVD	SVM	Acc = 99.00
[98] Gajic et al.	2014	Wavelet	Quadratic Classifier	Acc = 98.50
[99] Nabeel	2014	Statistical, Non-linear	Linear Classifier	Acc = 99.85
[100] Yatindra et al.	2014	Wavelet entropy	SVM	Acc = 90.00
[101] Jaiswal et al.	2015	EMD, Wavelet, Morphological filters	Fuzzy Clustering	PI = 98.03, QV = 23.82
[102] Rajaguru et al.	2015	Morphological filters	ANN	Acc = 98.33
[103] Bhattacharyya et al.	2015	Focal and non-focal, EWT	SVD, EM, MEM	Acc = 90.00
[104] Li et al.	2016	DD-DWT	LS-SVM	Acc = 99.36
[105] Li et al.	2016	Entropy	GA-SVM	AUC = 0.97
[106] Peker et al.	2016	DTCWT	CVNN	Acc = 100
[107] Riaz et al.	2016	EMD	SVM	Acc = 96.20
[108] Ghayab et al.	2016	SRS and SFS	LS-SVM	Acc = 99.90
[109] Upadhyay et al.	2016	DWT	LS-SVM	Acc = 100
[110] Kabir et al.	2016	Optimum allocation technique	LMT	Acc = 95.33
[111] Pippa et al.	2016	Time domain and frequency domain features	Bayesian Net	Acc = 95.00
[112] Jaiswal and Banka	2016	SpPCA and SubXPCA	SVM	Acc = 94.60
[113] Sharma and Pachori	2017	TQWT	LS-SVM + FD	Acc = 100
[114] Patidar et al.	2017	TQWT and Kraskov entropy	LS-SVM	Acc = 97.75
[115] Diykh et al.	2017	Weighted complex network combined with time domain features	LS-SVM	Acc = 98.00
[116] Li et al.	2017	MODWT and LND	RFC	Acc = 100
[117] Tiwari et al.	2017	Pyramid scheme for keypoint localization and LBP	SVM	Acc = 99.89
[118] Mursalin et al.	2017	ICFS	RFC	Acc = 100
[119] Shaikh et al.	2017	EMD	ANN	Acc = 96.10
[120] Kocadagli and Langari	2017	DWT and fuzzy relations	ANN	Acc = 99.90
[121] Torse et al.	2017	EMD	CSM-SVM	Acc = 96.40
[122] Sharma et al.	2018	MMSFL-OWFB-based KE	SVM	Acc = 100
[123] Tzimourta et al.	2018	Wavelet transform-based features	Random Forest Classifier	Acc = 95.00
[124] Sriraam et al.	2018	Teager energy feature	Supervised Backpropagation Neural Network	Acc = 96.66
[125] Sudalaimani et al.	2018	Sub-frequency band features	GRNN	Acc = 91.60
[126] Raghu and Sriram	2018	NCA	SVM	Acc = 98.80
[127] Li et al.	2018	GMM and GLCM features, RFE-SVM	SVM	Acc = 100
[128] Cooman et al.	2018	HRI features	SVM + Adaptive Heuristic classifier	EPsen = 83.30
[129] Li et al.	2018	WPT and KDE	LS-SVM	Acc = 99.60
[130] Cruz et al.	2018	ACC and EMG	SVM on CloudComputing Platform	Acc = 83.30
[131] Zhang et al.	2018	WPD, fDistIn	KNN	Acc = 98.33
[132] Feng et al.	2018	WPD	SVM	Acc = 98.67
[133] Tanveer et al.	2018	FAWT and entropy-based features	RELS-TSVM	Acc = 100
[134] Choudhury et al.	2018	XHST	KNN	Acc = 100
[135] Wani et al.	2018	DWT	ANN	Acc = 95.00
[136] Naser et al.	2019	DWT and approximation and abe entropies	SVM	Acc = 98.75
[137] Lamhiri and Shmuel	2019	Hurst exponent	k-ANN	Acc = 100
[138] Raghu et al.	2019	Sigmoid entropy	SVM	Acc = 100
[139] Wang et al.	2019	Symlet wavelet processing, and grid search optimizer	Gradient Boosting Machine	Acc = 96.10
[140] Bose et al.	2019	Multifractal detrended fluctuation analysis	SVM	Acc = 100
[141] Dalal et al.	2019	FAWT and FD	RELS-TSVM	Acc = 90.20
[142] Osman and Alzahrani	2019	SOM	RBFNN	Acc = 97.47
[143] Fasil O.K.; Rajesh R	2019	Time domain	Exponential Energy	Acc = 99.50
[144] Saminu et al.	2019	DWT, Entropies, Energy	SVM, FFANN	Acc = 99.00
[145] Mahjoub et al.	2020	TQWT, IMFs, MEMD	SVM	Acc = 98.78
[146] Raluca et al.	2020	DWT	ANN	Acc = 91.10
[147] Ozlem et al.	2020	Ensemble EMD	KNN	Acc = 97.00
[148] Khaled	2020	NA	Random Forest	Acc = 97.08

**Table 4:** Summary of reviewed works that used deep learning techniques.

Authors	Year	Features	Performance (%)
[163] Qi et al.	2014	MCC-based R-SAE model	EPsen = 100
[164] Thodoroff et al.	2016	CNN + RNN	EPsen = 85.00
[165] Johansen et al.	2016	CNN	AUC = 94.70
[166] Antoniades et al.	2016	CNN	EPacc = 87.51
[167] Lin et al.	2016	SSAE	EPacc = 96.00
[168] Achilles et al.	2016	CNN	AUC = 78.33
[169] Wei et al.	2016	Multichannel CNN	EPacc = 92.40
[170] Yuan et al.	2017	STFT-Mssda	EPacc = 93.82
[171] Gogna et al.	2017	Semi-supervised stacked autoencoder	EPacc = 96.90
[172] Ullah et al.	2018	P-1D-CNN	EPacc = 99.90
[173] Acharya et al.	2018	CNN	EPacc = 88.67
[174] Tjepkema-Cloostermans et al.	2018	CNN (1D and 2D) and/or LSTMs	EPspe = 99.90
[175] Yuvaraj et al.	2018	CNN	EPsen = 86.29
[176] Maria Hugle et al.	2018	CNN	EPsen = 96.00
[177] Thomas et al.	2018	CNN	EPacc = 83.86
[178] Hussein et al.	2019	LSTM + FC	EPspe = 100
[179] Emami et al.	2019	CNN	DR = 100
[180] Jang and Cho	2019	Dual deep neural network	EPsen = 100
[181] Haotian Liu	2019	CNN, LSTM, GRU	Acc = 0.96
[182] Rohan Akut	2019	WT-CNN	Acc = 99.40
[183] Thara et al.	2019	DNN	Acc = 97.21
[184] Turk et al.	2019	CNN	Acc = 93.6
[185] Akyol	2020	SEA	Acc = 97.17
[186] Rahib et al.	2020	Deep CNN	Acc = 98.67
[187] Zhou and Li	2020	Improved RBF	NA
[188] Ilakiyaselva et al.	2020	CNN	Acc = 98.50
[189] Gao et al.	2020	Deep CNN	Acc = 92.60
[190] Fabio et al.	2020	CNN	Acc = 98.82
[191] Kyung-Ok et al.	2020	CNN, FCNN, RNN	AUC = 0.993
[192] Wei Zhao et al.	2020	1D DNN	Acc = 99.52

## Abbreviations

ACD	acute consciousness disorder
ANN	artificial neural network
ApEn	approximate entropy
AR	autoregressive
CAD	computer-aided diagnosis
CCA	canonical correlation analysis
CD	correlation dimension
CDOC	chronic disorder of consciousness
CNN	convolutional neural network
DBF	deep belief network
DCNN	deep convoluted neural network
DOC	disorder of consciousness
DNN	deep neural network
DWT	discrete wavelet transform
EEG	Electroencephalogram
EESC	epileptic EEG signal classification
EOG	Electrooculogram
FDR	Fisher discriminant ratio
FA	firefly optimization
GMM	Gaussian mixer model
GRU	gated recurrent unit
HOS	higher-order spectra
HRS	hierarchical region splitting
ICA	independent component analysis
ICGA	integer coded genetic algorithm
IMF	intrinsic mode function
IoMT	internet of medical things
KNN	k-nearest neighbor
LLC	locally linear classification
LMS	least mean square
LMTS	long short-term memory
MCA	morphological component analysis
MCS	minimally conscious state
MRF	Markov random field
MRI	magnetic resonance imaging
NB	naive Bayes
NLMS	non-local means
PCA	principal component analysis
PD	Parkinson’s disease
PSD	power spectral density
PNN	probabilistic neural network
PSO	particle swarm optimization
RMS	root mean square
RLS	recursive least square
STFT	short time Fourier transform
SVM	support vector machine
SLSA	step-wise least square estimation algorithm
SRS	simple random Sampling
SSDA	stacked sparce density autoencoders
TCNN	temporal CNN
TGCN	temporal graph convolutional networks
TQWT	tunable Q-wavelet decomposition
UWS	unresponsive wakefulness syndrome
VS	vegetative state
WPE	wavelet packet entropy
WT	wavelet transform
WVD	Weiner–Ville distribution
